# Serum Activities of Paraoxonase 1 (PON1) in Predicting Liver Damage Among Patients Diagnosed With Hepatocellular Carcinoma: A Case-Control Study

**DOI:** 10.7759/cureus.46234

**Published:** 2023-09-29

**Authors:** Jyotchna D Bade, Vydehi Veeramalla, M Balachandra R Naidu, Danturty L Lalitha, Sarath Chandra Ponnada, Venkataramana Kandi

**Affiliations:** 1 Biochemistry, Great Eastern Medical School and Hospital, Srikakulam, IND; 2 Biochemistry, Siddipet Medical College, Siddipet, IND; 3 General Medicine, Great Eastern Medical School and Hospital, Srikakulam, IND; 4 Clinical Microbiology, Prathima Institute of Medical Sciences, Karimnagar, IND

**Keywords:** pon1, liver damage, liver function tests, paraoxonase, antioxidant, hepatocellular carcinoma

## Abstract

Introduction

Hepatocellular carcinoma (HCC) is one of the most common cancers in the world and unless diagnosed timely has limited options for treatment. Paraoxonase (PON) is a glycosylated protein that has been implicated in antioxidant and other biochemical functions. Paraoxonase 1 (PON1) is an esterase associated with high-density lipoprotein (HDL) particles. The present study was carried out to assess the PON1 activity and compare it with the standard liver function tests (LFTs) in assessing the predictability of liver damage among patients diagnosed with HCC.

Methods

This case-control study was carried out in the Department of Biochemistry attached to Great Eastern Medical School and Hospital, Srikakulam, Andhra Pradesh. Serum PON1 activities and LFTs like total bilirubin, direct bilirubin, alanine transaminase (ALT), aspartate transaminase (AST), alkaline phosphatase (ALP), total protein, and albumin were estimated in 30 patients diagnosed with HCC and 30 healthy persons. All the parameters were estimated using standard biochemical methods. The data was analyzed using GraphPad Prism version 6.0 (GraphPad Software, Inc). A probability (p) value <0.05 was considered to be statistically significant. Receiver operating characteristic curve (ROC) analysis was performed to assess the area under the curve (AUC) for accuracy, sensitivity, specificity, and diagnostic efficiency.

Results

The serum activities of PON1 had identical sensitivity (70%) to albumin (70%) and were superior to other tested parameters. Additionally, PON1 activities showed lower specificity (86.67%) than the other tested parameters. ROC analysis showed increased diagnostic efficacy (DE) of PON1 (DE=78.3%; p<0.0001) when compared with total bilirubin (DE=76.6%; p=0.0039), direct bilirubin (DE=74.9%; p=0.04), ALT (DE=73.30%; p=0.0006), and total protein (DE=71.6%; p=0.0005). However, the DE of PON1 was comparable with AST (DE=81.60%; p<0.0001), ALP (DE=79.9%; p<0.0001), and albumin (DE=83.30%, p<0.0001).

Conclusions

Serum activities of PON1 could be used as a diagnostic marker for assessing liver damage among HCC patients.

## Introduction

Hepatocellular carcinoma (HCC) is a primary cancer of the liver attributed to infection by hepatitis viruses (hepatitis B virus, hepatitis C virus) and non-infectious factors like alcoholism, non-alcoholic steatohepatitis (NASH), and microbial/fungal toxins [1,2]. Other conditions that could contribute to the development of HCC include α1 anti-trypsin deficiency, hereditary hemochromatosis, non-alcoholic fatty liver disease (NAFLD), glycogen storage diseases, tyrosinemia, genetic factors, age, gender, chemicals, hormones and nutrition [2]. The prevalence of HCC is higher in males than among females [3]. The incidence of HCC incidence varies widely in different parts of the world and is the sixth most common cancer [4]. According to Global Cancer Burden and Strategies for Cancer Control 2020, HCC accounts for 4.7% incidence of new cancer cases and 8.3% of deaths associated with HCC [5]. Liver cirrhosis precedes HCC in more than 80% of affected individuals [6].

The pathophysiology of HCC is complex, and the role played by reactive oxygen species (ROS) was previously investigated. It was noticed that ROS causes metabolic malfunction, oxidizes biological molecules, and results in deoxyribonucleic acid (DNA) damage thereby mediating carcinogenesis [7].

Human serum paraoxonase (PON) is a xenobiotic enzyme involved in the metabolism of organophosphorus compounds such as paraoxon, unsaturated aliphatic esters, aromatic carboxylic esters, neurotoxins, and insecticides. The PON enzyme appears in three forms including PON1, PON2, and PON3, and is coded on chromosome seven. They belong to a family of calcium-dependent hydrolases involved in antioxidant defense mechanisms [8-10].

Decreased PON1 enzyme activity was observed in alcoholic liver cirrhosis, chronic hepatitis, and NASH [8,11,12]. Several studies in the past have indicated that measuring PON1 enzyme activity could significantly improve the current efficacy of the laboratory’s evaluation of patients with suspected liver diseases like acute viral hepatitis, chronic alcoholic hepatitis, alcoholic cirrhosis, cirrhosis with portal hypertension, hereditary hemochromatosis and NAFLD [11,13-15].

The present study was carried out to assess the diagnostic utility of PON1 in comparison with conventional parameters in assessing liver damage among HCC patients.

## Materials and methods

This case-control study was carried out in the Departments of Biochemistry and Oncology of Great Eastern Medical School and Hospital (GEMSH), Srikakulam, Andhra Pradesh, India. The study recruited 30 HCC-diagnosed patients and an equal number of age- and sex-matched healthy persons as controls. The patient age was 57.33±9.85 years and the majority of them were males (27, 90%). The study was approved by the Institutional Ethics Committee of GEMSH, Srikakulam, Andhra Pradesh-532484 (107/IEC/GEMS&H/2023). Informed consent was obtained from all the participants. 

Inclusion criteria and exclusion criteria

Patients diagnosed with primary HCC with or without any associated co-morbidities like cirrhosis, hypertension, or diabetes mellitus were included in the study. Controls included in the study were healthy voluntary blood donors. The patients without HCC were excluded from the study.

Sample collection

Blood samples were collected by venipuncture with strict aseptic precautions. Five milliliters of blood were collected in a plain vacutainer. All the blood samples were centrifuged at 3000 rotations per minute for 10 minutes and the serum was separated. One part of the serum sample was taken for the analysis of Liver parameters like total bilirubin, direct bilirubin, alanine transaminase (ALT), aspartate transaminase (AST), alkaline phosphatase (ALP), total protein, and albumin, and another part was stored at -20ºC for PON1 analysis. Grossly hemolyzed and lipemic samples were excluded.

Biochemical parameters

The total bilirubin and direct bilirubin were estimated using the Diazo method of Pearlman et al. [16]. ALP, ALT, and ASP were analyzed using a method recommended by the International Federation of Clinical Chemistry and Laboratory Medicine (IFCC) [17]. Total proteins were estimated using the Biuret method introduced by Kingsley and modified by Henry [18]. The albumin was measured using bromocresol green (BCG) method as suggested by Doumas et al. [19]. All the parameters were executed on the Erba Chem 5 semi-autoanalyzer (Erba Diagnostics FZ-LLC; Miami Lakes, FL, USA).

Serum activity of PON1 was estimated using 4-nitrophenylacetate as substrate. It was acquired from Sigma-Aldrich (St. Louis, MO, USA). 250μl of one in 20 milliliters (ml) prediluted serum was mixed with 2ml tris hydrochloric acid (25mM at pH 7.4 and 25^ο^C) containing 1mM calcium chloride (CaCl_2_), 5% methanol, and 0.625mM of 4-nitrophenyl acetate. The rate of generation of 4-nitrophenol was determined at 402nm by calorimetric method in a spectrophotometer [20].

Statistical analysis

The data was analyzed using GraphPad Prism version 6.0 and the results were expressed as mean and standard deviation. A probability (p) value <0.05 was considered to be statistically significant. Receiver operating characteristic curve (ROC) analysis was performed to assess the area under the ROC curve (AUC) for accuracy, sensitivity, specificity, and diagnostic efficiency.

## Results

The patient age was 57.33±9.85 years and the majority of them were males (27, 90%). The parameters tested included PON1 and conventional liver function tests (LFTs). A comparison of the tested parameters among the cases and controls revealed significant variations in the parameters between the control group and the cases as shown in Table 1.

**Table 1:** Comparison of the tested parameters among cases and controls ALT: alanine transaminase; AST: aspartate transaminase; ALP: alkaline phosphatase; PON1: paraoxonase 1; SD: standard deviation; NA: not applicable; p: probability value (<0.05 is considered as statistically significant)

Parameters	Reference range	Controls (n=30) ( mean±SD)	Cases (n=30) (mean±SD)	p-value
Total bilirubin	0.1-1.2 mg/dL	0.82± 0.51	6.23±7.19	0.0001
Direct bilirubin	0.0-0.3 mg/dL	0.36±0.31	2.57±3.06	0.0002
AST	10-40 U/L	27.7±13.44	54.4±41.7	0.0015
ALT	10-40 U/L	24.31±14.36	76.47±42.2	< 0.0001
ALP	24-112 IU/L	70.63±15.01	246.2±312.7	0.0032
Total proteins	6.0-8.3 g/dL	7.21±0.96	6.09±1.77	0.0035
Albumin	3.2-5.0 g/dL	4.3±0.72	3.0±0.95	< 0.0001
PON1	NA	988±238	573.5±242.8	< 0.0001

The serum activities of PON1 had identical sensitivity (70%) to albumin (70%) and were superior to other tested parameters. Additionally, PON1 activities showed lower specificity (86.67%) than the other tested parameters. The best cut-off values of the tested parameters noticed from the study were total bilirubin (>2.2), direct bilirubin (>0.785), AST (>51.5), ALT (>34.7), ALP (>95.00), total protein (<6.265), albumin (<3.305), and PON1 (<738.5) as shown in Table 2.

**Table 2:** The sensitivity, specificity, and best cut-off values of the tested parameters ALT: alanine transaminase; AST: aspartate transaminase; ALP: alkaline phosphatase; PON1: paraoxonase 1; BCV: best cut-off value

Parameter/Variable	Total bilirubin (mg/dL)	Direct bilirubin (mg/dL)	AST (U/L)	ALT (U/L)	ALP (IU/L)	Total protein (g/dL)	Albumin (g/dL)	PON 1 (U/ml)
Sensitivity (%)	56.67	60	66.67	63.33	66.67	53.33	70	70
Specificity (%)	96.67	90	96.67	86.21	93.33	90	96.67	86.67
BCV	>2.2	>0.785	>51.5	>34.7	>95.00	<6.265	<3.305	<738.5

ROC analysis showed increased diagnostic efficacy (DE) of PON1 (DE=78.3%; p<0.0001) when compared with total bilirubin (DE=76.6%; p=0.0039), direct bilirubin (DE=74.9%; p=0.04), ALT (DE=73.30%; p=0.0006), and total protein (DE=71.6%; p=0.0005). However, the DE of PON1 was comparable with AST (DE=81.60%; p<0.0001), ALP (DE=79.9%; p<0.0001), and albumin (DE=83.30%, p<0.0001) as shown in Table 3.

**Table 3:** The accuracy and diagnostic efficacy of the tested parameters ALT: alanine transaminase; AST: aspartate transaminase; ALP: alkaline phosphatase; PON1: paraoxonase 1; DE: diagnostic efficacy; p: probability value (<0.05 is considered as statistically significant)

Parameter/statistics	Total bilirubin	Direct bilirubin	AST	ALT	ALP	Total protein	Albumin	PON 1
AUC	0.7161	0.6522	0.8694	0.7609	0.8428	0.7617	0.8761	0.8772
DE (%)	76.6	74.9	81.6	73.3	79.9	71.6	83.3	78.3
p-value	0.0039	0.04	<0.0001	0.0006	<0.0001	0.0005	<0.0001	< 0.0001

## Discussion

The results of this study indicate the potential utility of estimating PON1 activities among HCC patients. Additionally, PON1 activities were comparable with conventional LFTs. The DE of PON1 was found to be similar and moderately superior to the LFTs. Therefore, it is evident that PON1 estimations could potentially be helpful in evaluating liver damage and predicting disease progression among HCC patients. 

Liver diseases often involve inflammatory processes and elevated levels of cytokines and chemokines in the blood stream are commonly associated with these conditions. Cytokines and chemokines are molecules that regulate immune responses and their increased presence indicates ongoing inflammation in the liver. Furthermore, oxidative stress plays a crucial role in the development and advancement of liver diseases. Oxidative stress arises from an imbalance between the production of ROS and the body’s ability to neutralize them with antioxidants. Due to the liver’s involvement in metabolizing various substances, it becomes susceptible to oxidative stress. Chronic inflammation and excessive oxidative stress can lead to cellular damage and contribute to the progression of liver diseases [21]. In chronic liver diseases, oxidative stress influences the pathophysiological changes leading to liver cirrhosis and HCC.

Chronic liver diseases exhibit common biochemical changes, regardless of their underlying causes, which are influenced by oxidative stress. This oxidative stress is a result of mitochondrial abnormalities, leading to alterations in lipid and lipoprotein metabolism, fat accumulation, increased cytokine synthesis, and extracellular matrix deposition. One enzyme known for its protective role against oxidative stress is PON1. Hence, it is reasonable to expect a connection between PON1 and liver damage [22].

Ferré et al. conducted a study on rats with carbon tetrachloride-induced fibrosis to investigate early biochemical changes related to increased lipid peroxidation and liver damage [23]. They explored the relationship between hepatic microsomal PON1 activity, lipid peroxidation, and disease progression using this experimental model. Additionally, the researchers examined how a dietary supplementation of zinc, known for its antioxidant and anti-fibrogenic effects, influenced these processes. Their findings indicated a decrease in PON1 activity and an increase in lipid peroxidation in rats exposed to carbon tetrachloride, but the addition of zinc regularized PON1 activity and lipid peroxidation. These results suggested that PON1 might play a crucial role in defending liver health against free radical production.

In their subsequent study, Ferré et al. observed a patient's liver disease and found a correlation between enhanced PON1 protein expression in the liver, increased serum soluble Fas (a marker of anti-apoptosis) concentration, and reduced Fas-positive cell clusters (markers of apoptosis) and parenchymal cell DNA fragmentation. The patients with liver disease also exhibited higher serum PON1 concentration. These findings suggested a potential link between PON1 and the anti-apoptotic capability of HDL molecules, potentially attributed to PON1’s ability to protect lipoproteins against oxidation [24].

A study done by Sun et al. focused on glucosylated PON1. The research specifically examined a subgroup of fucosylated and sialylated proteins that showed significant change in HCC patients. PON1 was identified as one of the core nodes in the protein-protein interaction related to lipid metabolism, suggesting a potential role for PON1 in regulating lipid metabolism in HCC patients. Their study results indicated that the protein expression of PON1 was reduced by half in both liver cirrhosis and HCC. Interestingly, the levels of fucosylation and sialylation of PON1 increased significantly in liver cirrhosis, and this increase was even more dramatic in early HCC. The observed rise in glycosylated PON1 in HCC might be attributed to the upregulation of corresponding glycosyl transferases, potentially linked to cancer-induced lipid metabolism disorder and a potential impairment in the anti-lipid peroxidase and anti-oxi radical abilities of PON1 [25].

The available studies have proposed several mechanisms to explain the decrease in serum PON1 activity in chronic liver diseases. Firstly, these patients exhibit increased free radical production, and it has been reported that PON1 is inactivated after hydrolyzing lipid peroxides. Secondly, alterations in HDL structure and composition can impact PON1 activities. Thirdly, the reduced PON1 activities may be a consequence of altered PON1 synthesis by the liver [26-28].

A recent study evaluated the efficacy of serum PON1 activities in predicting microvascular invasion among HCC patients. This large-scale study involved more than 700 HCC patients, the results of which suggest that PON1 measurement among HCC patients may be beneficial in the management of patients and assess prognosis [29]. 

There has been an increased interest in reference to the role played by PON1 in the development of human diseases. Human and animal experimental studies have suggested the potential role played by PON1 in several cardiovascular and central nervous system disorders including atherosclerosis and Alzheimer's disease, respectively [30].

Study limitations

This study was carried out as a case-control study and included only 30 HCC patients and an equal number of controls. Therefore, the results obtained from this study do not necessarily indicate a population scenario. Additionally, further large-scale studies may be warranted to confirm these results.

## Conclusions

Liver diseases are gradual in progression and effective clinical management is the mainstay for patient management. Patients suffering from HCC undergo liver changes that are essentially detected using various laboratory methods. The assessment of liver abnormalities is generally carried out using conventional LFTs. However, there are multiple parameters that are assessed, and the physicians treating HCC patients are required to carefully evaluate the changes in these parameters. Alternatively, the PON1 activities may be used to evaluate the extent of liver damage. The results from this study indicate that the serum activities of PON1 could be used as a diagnostic marker for assessing liver damage among HCC patients.
